# Expression of antimicrobial host defence peptides in the central nervous system during health and disease

**DOI:** 10.1093/discim/kyac003

**Published:** 2022-07-26

**Authors:** Katie J Smith, Emily Gwyer Findlay

**Affiliations:** Centre for Inflammation Research, University of Edinburgh, 47 Little France Crescent, EH16 4TJ, Edinburgh, UK; Centre for Inflammation Research, University of Edinburgh, 47 Little France Crescent, EH16 4TJ, Edinburgh, UK

**Keywords:** brain, central nervous system, host defence peptides, neurodegeneration, cathelicidin, defensins, dermcidin, hepcidin

## Abstract

Antimicrobial host defence peptides (HDP) are critical for the first line of defence against bacterial, viral, and fungal pathogens. Over the past decade we have become more aware that, in addition to their antimicrobial roles, they also possess the potent immunomodulatory capacity. This includes chemoattracting immune cells, activating dendritic cells and macrophages, and altering T-cell differentiation. Most examinations of their immunomodulatory roles have focused on tissues in which they are very abundant, such as the intestine and the inflamed skin. However, HDP have now been detected in the brain and the spinal cord during a number of conditions. We propose that their presence in the central nervous system (CNS) during homeostasis, infection, and neurodegenerative disease has the potential to contribute to immunosurveillance, alter host responses and skew developing immunity. Here, we review the evidence for HDP expression and function in the CNS in health and disease. We describe how a wide range of HDP are expressed in the CNS of humans, rodents, birds, and fish, suggesting a conserved role in protecting the brain from pathogens, with evidence of production by resident CNS cells. We highlight differences in methodology used and how this may have resulted in the immunomodulatory roles of HDP being overlooked. Finally, we discuss what HDP expression may mean for CNS immune responses.

## Introduction

Antimicrobial host defence peptides (HDP) are a family of short peptides with diverse sequences, produced both constitutively and in response to bacterial, viral, and fungal infections. They are expressed in multiple tissues and fluids throughout the body, including serum, saliva, semen, sweat, lung, intestine, and skin [1–6]. Cells that are well documented to produce HDP include neutrophils, mast cells, macrophages, Paneth cells, and mucosal epithelium [7–10]. Features of abundant HDP are shown in Table 1.

**Table 1:** Features of antimicrobial peptides expressed in the CNS. PDB—reference code for entry in the protein data bank. Created with BioRender.com.

Peptide	Structure	Size (# residues, human form)	Charge
Cathelicidin	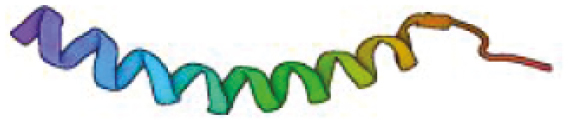 Human *CAMP* (LL-37) PDB: 2KSO Wang., 2008	37	Cationic +6
Beta defensin 1	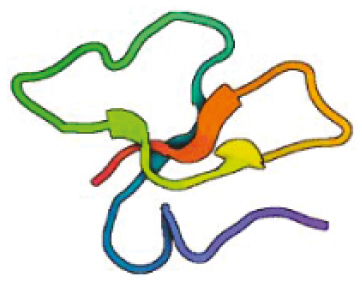 Human *DEFB1* PDB: 1KJ5 Schibli et al., 2001	36	Cationic +4
Beta defensin 2	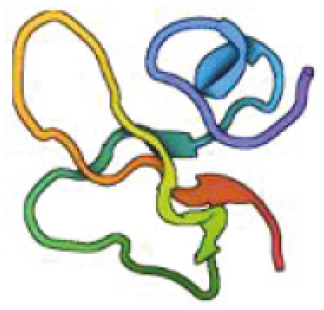 Human *DEFB4* PDB: 1FQQ Sawai et al., 2001	41	Cationic +6
Dermcidin	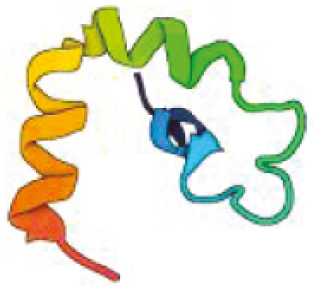 Human *DCD* PDB: 2KSG Jung et al., 2010	48	Anionic −5
Hepcidin	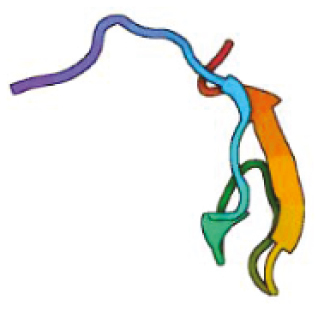 Human *HAMP* PDB: 2KEF Jordan et al., 2009	25	Cationic +3

**Figure 1:**
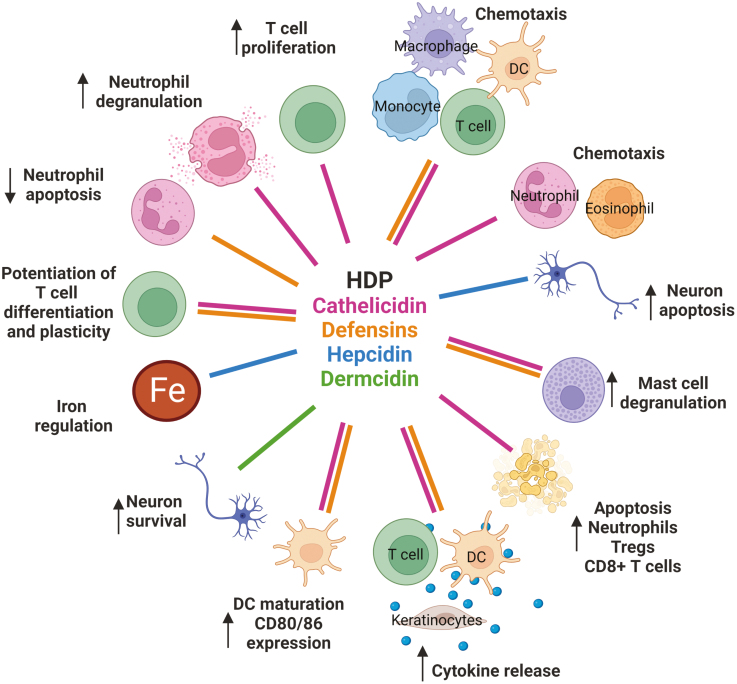
Known immunomodulatory effects of host defence peptides. Created with BioRender.com.

In addition to their antimicrobial capacities, HDP are potent immunomodulators. In particular, defensins, and cathelicidins can alter dendritic cell activation and differentiation [11–14], skew T-cell differentiation [13, 15], halt macrophage mRNA translation [16], and chemoattract immune cells including monocytes and T cells [17] (Fig. 1). As such, their production not only drives defence against infiltrating infection but also substantially alters immunity during infectious and inflammatory disease (HDP immunomodulatory capacities are summarized in Fig. 1).

**Figure 2:**
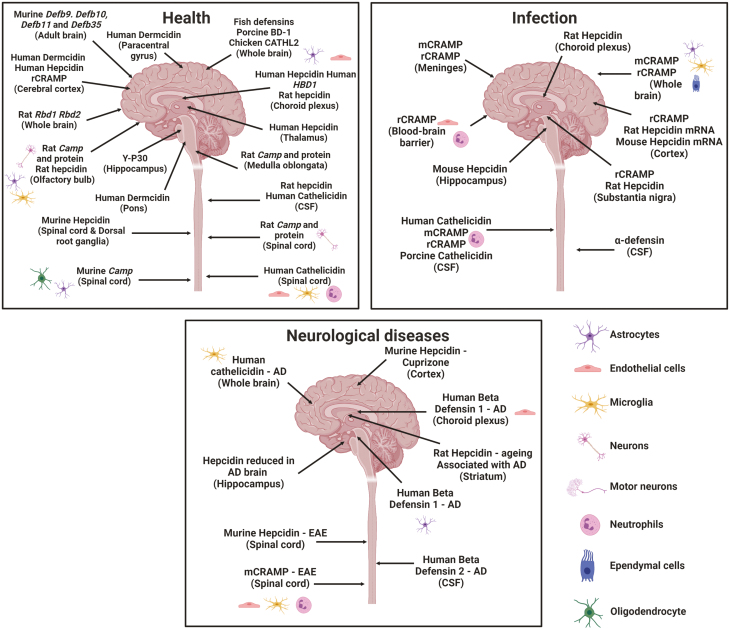
The expression of host defence peptides across the central nervous system in health, infection and neurological disease. Created with BioRender.com.

The expression of a variety of peptides has been widely described in many tissues, with a particular abundance in the intestine [18, 19], lung [20, 21], and skin [22–24]. However, one exception has been the central nervous system (CNS). The production of HDP by resident and infiltrating cells of the CNS, and their capacity to modulate immunity there, has not previously been reviewed, despite mounting evidence that HDP are indeed present at this site (Fig. 2 and Table 2). Here, we review the expression and discuss the potential functions of HDP throughout the nervous system of multiple species in health, infection, and neurodegenerative disease.

**Table 2:** 

Peptide	Expression in the CNS	Cellular source	References
Cathelicidin	Whole brain, olfactory bulb, medulla oblongata, spinal cord, hippocampus, striatum, cerebellum, dorsolateral prefrontal cortex, anterior cingulate cortex, meninges, CSF	Microglia, astrocytes, motor neurons, Purkinje cells, olfactory bulb neurons, dorsolateral prefrontal cortex, anterior cingulate cortex, BBB endothleial cells, infiltrating neutrophils, meningeal cells, neuronal cell lines	Bals et al. (1998), Bergman *et al.* (2005), van Dijk *et al.* (2005), Bergman *et al.* (2006), Brandenburg *et al.* (2009), Brandenburg *et al.* (2008). Brandenburg *et al.* (2010), Lewis *et al.* (2014), Lee *et al.* (2015), Byfield *et al.* (2011), de Buhr *et al.* (2017), Postolache *et al.* (2020), Hassel *et al.* (2018)
Beta defensin 1	Whole brain, choroid plexus, hippocampus, spinal cord	Neuronal cells, astrocytes	Huttner *et al.* (1997), Nakayama *et al.* (1999), Hiratsuka *et al.* (2001), Froy *et al.* (2007), Maxwell *et al.* (2003), Morrison *et al.* (2003), Zhang *et al.* (1998), Hao *et al.* (2001), Schluesener and Meyermann (1995), Williams *et al.* (2013), Fleming *et al.* (2006)
Beta defensin 2	Whole brain (low levels)	Immortalized brain capillary endothelial cells, neuronal cells	Huttner *et al.* (1997); Hiratsuka *et al.* (2001), Froy *et al.* (2007), Maxwell *et al.* (2003), Morrison *et al.* (2003), Tiszlavicz *et al.* (2011), Hao *et al.* (2001), Soman *et al.* (2009)
Dermcidin	Pons, paracentral gyrus, locus ceruleus, nucleus raphe pontis, substantia nigra, lateral hypothalamic nuclei	Unknown	Porter *et al.* (2003)
Hepcidin	Whole brain, choroid plexus, cortex, thalamus, hippocampus, striatum, substantia nigra, choroid plexus, spinal cord, dorsal root ganglia	Astrocytes, epithelial cells of choroid plexus, neurons, immortalized mouse microglia	Hanninen *et al.* (2009),, Hanase *et al.* (2020), Raha-Chowdhury *et al.* (2015), Zechel *et al.* (2006), Pandur *et al.* (2019), Urrutia *et al.* (2013); Wang *et al.* (2008), Zarruk *et al.* (2015), and Varga *et al.* (2018)

## Cathelicidin

The short cationic peptide cathelicidin has multiple well-defined antimicrobial and immunomodulatory roles. Its expression has been described in many tissues such as the skin [25], intestine [6], airways [21], and reproductive system [26, 27]. Many cell types can produce cathelicidin; it is stored at a high concentration in neutrophil secondary granules and can also be produced by monocytes, macrophages, mast cells, adipocytes, and some T-cell subsets [10, 28–30]. It has direct and indirect anti-bacterial, anti-viral, and anti-fungal action [31–33], with direct killing observed against the respiratory syncytial virus [32], many bacterial species including *Escherichia coli* and *Streptococcus pneumoniae* (reviewed in [34]) and fungal species including *Candida albicans* [35]. As a consequence, mice lacking cathelicidin are more susceptible to a variety of infections [31, 36, 37].

Cathelicidin is also a powerful immunomodulator and understanding its impact is a burgeoning field of immunology. For example, it can mature dendritic cells and up-regulate their T cell priming capacities [11, 13, 14], chemoattract innate and adaptive immune cells, enhance T-cell survival and Th17 differentiation [15, 17, 38], and induce re-epithelialization and re-endothelialization following damage [39, 40]. It is therefore important to understand whether cathelicidin is expressed in the CNS, and whether its immunomodulatory roles are important at that site.

### Health

During steady state, cathelicidin is expressed in the human CNS: in patients with conditions that do not clinically alter cerebrospinal fluid (CSF) composition (idiopathic cephalgia, ischialgia due to discopathy, and idiopathic facial nerve palsy) cathelicidin has been detected in the fluid (in the range 0.01–0.07μM) [41]. Interestingly, this is similar to the mean cathelicidin concentration in healthy plasma, which is 0.07 μM (*n* = 58, SD = 0.20) [7]. Dot blot hybridization demonstrated cathelicidin expression in whole healthy human brain homogenate to be, strikingly, at similar levels to the colon and the lung [21]. A more recent study that generated a single cell atlas of the human spinal cord showed that cathelicidin mRNA was expressed—albeit at low concentrations—in some astrocyte and oligodendrocyte populations [42]. Finally, recent work from our laboratory has shown that cathelicidin is expressed at the protein level in human post-mortem brain tissue from donors who did not die from neurological causes [43]. In these samples, cathelicidin was expressed by neutrophils, CD68^+^ microglia/macrophages, and endothelial cells, which to our knowledge is the first demonstration of these cells expressing cathelicidin in a healthy brain [43]. Thus, cathelicidin mRNA and protein are expressed during homeostasis in the human CNS.

Cathelicidin is also present in the central nervous systems of rodents, birds, and small mammals. mRNA encoding rat cathelicidin was detected in the olfactory bulb, medulla oblongata, and spinal cord of healthy brains [44]. In this study, rat cathelicidin was also detected in primary cell cultures of the hippocampus, striatum, cerebellum, and medulla oblongata. Furthermore, CMAP27, a chicken cathelicidin-like antimicrobial peptide, is expressed at the mRNA level in the brain [45]. This data suggests cathelicidin expression is conserved across different species in the steady-state CNS. In contrast, cathelicidin mRNA was not detected in healthy mouse brains using northern blot analysis [46], nor in our examinations of the healthy mouse spinal cord [43], making mice unusual in their lack of CNS cathelicidin expression.

### Infection

As HDPs are critical in the innate immune response, it is likely they are important innate responders to infection in the CNS. Not surprisingly, cathelicidin levels are elevated in human CSF during infection. During active bacterial meningitis, cathelicidin is increased up to 0.02 μM in the CSF of patients, compared to 0.0025 μM in the healthy controls of this study [47]. Cathelicidin concentration positively correlates with CSF bacterial count [48] and with CSF white cell counts [49]. Similarly, CSF cathelicidin was elevated in children with tuberculous meningitis compared to healthy controls [50]. Moreover, in human CSF cathelicidin levels are increased in tuberculosis meningitis-positive HIV patients compared to tuberculosis meningitis-182 negative HIV patients [51].

Experiments to determine the cellular source of CNS cathelicidin have determined a surprisingly wide range of resident and infiltrating cells able to produce it. During *Neisseria meningitidis* infection, rat cathelicidin production increased in endothelial cells and infiltrating neutrophils in the meninges, as measured by immunohistochemical analysis [52]. It was also detected in the brains of rats 12, 22, and 39 h after *Streptococcus pneumoniae* infection [53] and can be produced by microglia and astrocytes following *Pneumococcal meningitis* infection [47]. Moreover, neutrophil extracellular traps coated with high concentrations of cathelicidin are released in the CSF following bacterial meningitis infection in rats [54] and *Streptococcus suis* infection in piglets [55]. In mice, cathelicidin is expressed in the meninges and brain parenchyma after pneumococcal infection and mice lacking cathelicidin have increased mortality following infection with *Streptococcus pneumonia* to induce meningitis [56]. Moreover, cathelicidin is expressed in the meninges and brain parenchyma of mice after pneumococcal infection.

Therefore, in many species cathelicidin is upregulated during CNS infection and can be produced by glial cells, endothelial cells, and infiltrating neutrophils. As cathelicidin has potent anti-bacterial and anti-viral activity, it is highly likely that cathelicidin will be involved in clearance of CNS invading pathogens. However, cathelicidin also has powerful immunomodulatory roles. In one study, it promoted signal transduction in glial cells leading to IL-6 production, in a manner dependent on ERK1/2, p38 MAPK, and NFκB [57]. Its role in glial cell function appears complex, with some evidence that it may play a regulatory role—for example cathelicidin-knockout glial cells have a pronounced pro-inflammatory response following meningitis infection [58]. Unpicking how regulation of immune responses is separate from cathelicidin’s direct anti-endotoxic and anti-inflammatory effects will take considerable work.

### Neurodegeneration

HDP-secreting neutrophils migrate into the CNS during neuroinflammation. Neutrophil infiltration into the spinal cord and brain tissue has been observed in mouse models of Alzheimer’s disease (AD) [59, 60] and multiple sclerosis (MS) [61, 62]. Importantly, cathelicidin-positive neutrophils are present in the spinal cord in experimental autoimmune encephalomyelitis (EAE), a mouse model of MS. Depletion of neutrophils or cathelicidin attenuates the development of EAE [43, 60, 62–64] and in AD models this can improve cognitive decline [65].

In humans, neutrophils are present in the CSF and in active lesions of neuromyelitis optica patients [66]. Moreover, it has been shown that neutrophils release extracellular traps (NETs) in the brain parenchyma of AD patients [60]. MS peripheral blood neutrophils have increased activation markers and enhanced degranulation [67]. As cathelicidins, as well as other HDPs including defensins, are secreted during degranulation and are present on NETs, these studies suggest HDP could have a functional role during neutrophil effector mechanisms in these diseases.

Immunohistochemistry in post-mortem brains from patients with Alzheimer’s disease (AD) showed cathelicidin expression to be increased in microglia and astrocytes compared to healthy donor brain samples [68]. We have recently shown that cathelicidin is expressed in active lesions in the brains of patients with MS and in the spinal cords of mice undergoing the model of MS experimental autoimmune encephalomyelitis (EAE) [43]. In both cases, the majority of cathelicidin was released by neutrophils, but it was also seen expressed by microglia and by endothelial cells. Not only is cathelicidin expressed in the CNS but also it plays a key role in promoting damaging inflammation such that mice lacking the peptide are resistant to developing severe EAE. Therefore, during MS cathelicidin plays a role in potentiating harmful immune responses.

## Defensins

Defensins are cationic peptides with a characteristic series of cysteine residues, which form an antiparallel β-sheet structure. There are two classes, α- and β-defensins [69], which have broad anti-microbial, anti-viral, and anti-fungal activity [70]—for example they have demonstrated activity against *Staphylococci* [71], herpes simplex virus [72], influenza virus [73], HIV [74], and *Candida albicans* [75].

α-defensins are stored in neutrophil primary granules at high concentrations and are released from Paneth cells in the intestine [69, 76]. They have been associated with CNS infections previously, being elevated in the CSF of children with bacterial meningitis (with a median of 23μg/ml in infected subjects and with no control subjects having detectable defensins in the CSF) [77]. Using LC–MS/MS analysis and ELISA techniques, α-defensin 1 was detected in the CSF of patients with West Nile neuroinvasive disease and non-WNV CNS infections [78]. It is expected that these CSF defensins are being released from neutrophils, and there are no published papers to our knowledge showing the expression of α-defensins by CNS-resident cells. To examine this further, we have carried out mining of published sequencing datasets of neurons, astrocytes, microglia, and oligodendrocytes. None of these datasets showed any expression of the alpha defensin genes *Defa1, Defa2, Defa3, Defa4, Defa5, or Defa6.* It is likely therefore that they are only being released by neutrophils, and also that the methods we use currently have led to the impact and quantity of this being underestimated (see note at end on Methodology). For the remainder of this review, we will focus on β-defensins.

β-defensins are not only associated with infection but also have significant immunomodulatory roles. Their production is increased in monocytes by LPS but also by IFN-γ and vitamin D [9, 79] indicating inflammation-related and not only infection-related mediators can switch on defensin expression.

Defensins have multiple roles in many immune cells. For example, human β-defensin 3 modulates TLR4 signalling [80], chemoattracts monocyte/macrophages [81], alters macrophage differentiation and increases their IL-4 production [82], and enhances dendritic cell responses to bacterial DNA in a TLR-9 dependent manner [12]. Mouse βD-14 switches CD4+ CD25− T cells into regulatory T cells inducing expression of FOXP3 and CTLA-4 [83]. β-defensins can also be anti-inflammatory, as hBD3 in the presence of LPS inhibits IL-6 and TNF-α accumulation in the human myelomonocytic cell line THP-1 and peripheral blood monocytes derived macrophages [84]. As a family of peptides, therefore, they have the varied immunomodulatory capacity that affects both innate and adaptive immune cells.

### Health

In humans, early work analyzing the widespread expression of hBD-1 in the CNS showed it is not expressed in the mRNA isolated from the brain (although the specific region was not specified) [85]. Further, more specific, a study of frozen brain tissue of patients without CNS disorders showed that hBD-1 mRNA is expressed in the choroid plexus but not in the cerebral cortex, cerebellum, pia mater, or leptomeningeal vessels [86]. In this study, hBD-2 was not detected in any brain regions examined. The first study used northern blot analysis to assess hBD-1 expression, whilst the latter used RT-PCR analysis. This highlights the requirement for multiple experimental techniques to fully understand HDP expression within tissues; as detailed below (see ‘A note on methodology’), detection of HDP is sometimes difficult. Moreover, this data suggests in humans there is a regional expression of hBD-1 as well as differential expression of individual defensins. The choroid plexus serves as an interface between the CNS and the periphery, is a niche for resident immune cells, and has been shown to be the site of T-cell stimulation in the CNS [87–90]. As the choroid plexus was the only site in the brain that expressed hBD-2 in this study, it is possible that hBD-2 expression at this site is part of the immune surveillance of the CNS by immune cells. Further understanding of the function of human defensins in the CNS during steady-state may help elucidate these differences.

β-defensins are expressed in the brain of a wide variety of species. Porcine BD-1 is expressed in the brain of 4–5-week-old pigs [91]. Studies using RT-PCR show that *Rbd1* and *Rbd2* are expressed at low levels in the rat brain [92, 93] and the bovine β-defensin is expressed in the meninges and choroid plexus of healthy adult cows [94]. Likewise, *Defb9*, *Defb10*, *Defb11,* and *Defb35* are expressed in the adult mouse brain and *Defb10*, *Defb11,* and *Defb35* are expressed in the neonate [95, 96]. In health, the levels of the beta-defensin homologue, *gcdefb1,* in Chinese grass Carp showed the highest expression in the brain compared to other tissues [97]. In other healthy fish such as mandarin [98] and orange-spotted grouper [99], β-defensin transcripts are present but are expressed at low levels in the brain. The duck β-defensin-2 homologue is also expressed at low levels in the brain in healthy ducklings [100]. In addition, members of the β-defensin family are differentially expressed; in rainbow trout, for example, four novel members of the family were identified but only omBD-3 was expressed at low levels in the brain [101], and in the blunt snout bream maBD-2 was expressed in the brain, but not maBD-2 [102].

Thus, defensins are expressed widely and are conserved across different species. It is likely that the expression of defensins during a steady state plays a role in immune surveillance and may have important functions in regulating immune responsiveness of resident CNS cells. However, whether the expression of different defensins, and their expression in different brain regions possess different functions is unclear and warrants further investigation.

### Infection

Surprisingly, there are very few studies investigating the expression of β-defensins in the CNS following infection. One study demonstrated the expression of hBD-2 mRNA and protein by immortalized human brain capillary endothelial cells after *Chlamydophila pneumoniae* infection [103]. Another study demonstrated that stimulating astrocytes cell cultures with LPS, IL-1β, or TNF-α—to model infection—stimulated the production of hBD1 and hBD2 mRNA and protein, whilst meningeal fibroblasts and microglia were only able to express hBD1 mRNA [104]. These results suggest a possible role for hBD2 in early immune responses of the brain.

### Neurodegeneration

It has been suggested that β-defensins could play a role in the neuroimmune function and during neurodegeneration [105]. Williams and colleagues hypothesize that conditions such as hyperglycaemia and increased insulin resistance, which are present in many neurological conditions, may alter defensin expression; for example, high glucose induces hBD2 and hBD3 mRNA expression from primary epithelial cells *in vitro.* In addition, they suggest that abnormal expression of β-defensins could contribute to loss of AMP-induced regulation of dendritic cells and chronic inflammation.

Similarly, levels of hBD-2 were significantly elevated in the sera and in the CSF of patients with AD compared to age-matched controls [106]. In addition, hBD-1, but not hBD-2 or h-BD-3, is present within hippocampal astrocytes as well as in neurons and the choroid plexus and is increased in patients with AD [107].

## Dermcidin

Dermcidin is a non-classical HDP that shares no homology with other known antimicrobial peptides [3]. It is secreted constitutively by eccrine sweat glands at a concentration of ~1–10 µg/ml, and is transported to the epidermal surface [3]. Dermcidin is proteolytically cleaved into an active form which is 47 amino acids in length [3] and, unlike other positively charged HDPs, its charge is −5 [3]. It has potent antimicrobial activity, contributing to the immune defence of the skin [108]. Broad spectrum activity against several different pathogens has been described such as *Staphylococcus epidermidis, Pseudomonas aeruginosa, Pseudomonas putida,* methicillin-resistant *S. aureus, Listeria monocytogenes,* and *Salmonella typhimurium* [109–111]. This peptide was originally identified in humans [3], and interestingly dermcidin has no homologue in rodents or other mammals except for primates [108].

### Health

Dermcidin is expressed in the uninfected brain; northern blot analysis showed dermcidin expression specifically in the pons of healthy adult and foetal human brains, with low expression also noted in the paracentral gyrus of the cerebral cortex [112].

An AP-Dermcidin fusion protein showed strong binding to neurons in the locus ceruleus, nucleus raphe pontis, substantia nigra, and the lateral hypothalamic nuclei and weak binding to almost all neurons in the healthy human adult brain [112]. The authors suggest that dermcidin could be acting as a survival factor for neurons that have increased sensitivity to reactive oxygen species [112]. Similarly, Y-P30 (the first 30 amino acids of the dermcidin precursor protein [108]), has been detected in neonatal rats and human foetal brains in the neocortex and hippocampus. Under oxidative stress conditions, Y-P30 has been shown to promote the survival of retinoblastoma cells, hepatocellular carcinoma cell line HuH7, and the prostate cancer cell line PC-3M [113, 114].

### Infection

Dermcidin has broad antimicrobial activity against many bacteria including *Staphylococcus, Listeria* and *Salmonella* species, but to our knowledge its activity during CNS infection has not been investigated. High dermcidin production is exclusive to the pons of the healthy human brain [112]. As the pons has an active relationship with the periphery [115], expression of dermcidin in this brain region could be an important mechanism for innate defence against infection. However, it remains to be clarified whether dermcidin is expressed in the non-inflamed brain at the protein level. Alternatively, it is possible that dermcidin has more important neuro-modulatory and neurological maintenance functions, some of which are yet to be discovered.

### Neurodegeneration

In serum samples from AD patients, dermcidin expression was increased and it was suggested as a potential biomarker for disease [116], but other studies are so far not available.

As discussed above, the dermcidin precursor protein Y-P30 can promote neuronal survival [112–114]; however, this has not been investigated under neurodegenerative conditions. Other research has demonstrated that Y-P30 also promotes neurite outgrowth from thalamic and cerebellar neurons and is neuroprotective following optic nerve damage [113, 114, 117–119]. Therefore, as Y-P30 can be neuroprotective during injury, perhaps if the dermcidin expression is dysregulated this could lead to abnormal neuronal maintenance and potential neurodegeneration. However, testing this hypothesis will require significant further study.

## Hepcidin

Hepcidin is a cysteine-rich cationic peptide produced in the liver, which has multiple immunomodulatory and antimicrobial activities including against *Candida albicans, Aspergillus fumigatus, Escherichia coli, Staphylococcus aureus, Staphylococcus epidermidis,* and group B *Streptococcus* [120]. Mutations in *HAMP* have been identified in patients suffering from hereditary hemochromatosis [121]. This role appears to be unrelated to its action as an antimicrobial peptide, as these patients do not have increased susceptibility to infections, but to its immunomodulatory action. Hepcidin also has many non-immunomodulatory functions throughout the brain, specifically its role with regard to iron homeostasis which has been reviewed extensively elsewhere [122, 123].

### Health

Hepcidin mRNA is present in the uninfected human brain with relatively high expression found in the cortex, cerebellum, thalamus, medulla oblongata, and hippocampus, with the highest expression in the cortex and thalamus [124]. Hepcidin has also been detected within granular structures of astrocytes and in the epithelial cells of the choroid plexus [125].

It is also widely distributed in healthy mouse and rat brains and spinal cord. In mice, hepcidin-1 and hepcidin-2 are present in the CNS and immunohistochemistry of hepcidin-1 was observed in many regions such as the olfactory bulb, cortex, hippocampus, amygdala, thalamus, hypothalamus, mesencephalon, cerebellum, pons, spinal cord, as well as in dorsal root ganglia of the peripheral nervous system [126]. The same study showed using immunohistochemistry that hepcidin-1 was expressed by neurons and glia cells in the adult mouse CNS [126]. The authors failed to show a similar distribution of hepcidin by *in situ* hybridization which they suggest is because the mRNA signal is below the detection limit [126]. As hepcidin is an important iron regulator in the periphery, the authors suggest it is possible the same is occurring in the CNS.

In rats, Raha-Chowdhury showed by RT-PCR that hepcidin mRNA was expressed at low levels throughout the brain, while *in situ* hybridization showed hepcidin mRNA was restricted to the endothelium of blood vessels and the choroid plexus [127]. Hepcidin protein was expressed in the sub-ventricular zone, cortex, and the CSF, and associated with the epithelial cells of the choroid plexus, endothelial cells, pericytes, and astrocytes. The authors suggested that due to their observation that hepcidin is expressed in all layers of the BBB blood vessel walls and pericytes, peripheral hepcidin could also be crossing the intact BBB into the CNS [127].

### Infection

There is increasing evidence that hepcidin acts as an antimicrobial agent in the CNS. Intravenous LPS injection in rats significantly increases hepcidin mRNA and protein expression in the cortex and the substantia nigra but not in the striatum or hippocampus [128, 129]. Similarly, peripheral administration of LPS in mice increases hepcidin gene expression in the choroid plexus [130]. It is not clear whether the regional specificity of hepcidin upregulation is physiologically functional, or if it is due to differences in the sensitivity of methods used, as other studies have failed to detect hepcidin mRNA in the cortex [127]. This LPS-induced expression was mediated through the IL-6/STAT3 pathway in the mouse cortex and hippocampus [131]; in IL-6 KO mice, hepcidin mRNA levels in these regions are significantly reduced [131]. Another study showed that this same pathway also occurs in the choroid plexus during ageing in rats [132].

Interestingly, there may be cell-specific regulation of LPS-mediated expression of hepcidin in the brain. Hepcidin mRNA is expressed at a much lower level in neurons than in astrocytes, and treatment with IL-6 in IL-6 KO astrocytes and neurons resulted in higher increased expression in astrocytes compared to neurons [133]. There is also evidence for cell-cell communication in this pathway, with LPS increasing hepcidin expression in neurons only when in culture with microglia, indicating microglia were the source of IL-6 (131). Thus, bacterial agents directly activate inflammatory signalling pathways which lead to the production of hepcidin in CNS resident cells—this is likely part of the CNS immune response.

Hepcidin is an important modulator of iron homeostasis and acts as a regulator of cellular iron release by binding to ferroportin 1 [134]. Its production in immortalized mouse microglia cells increases after stimulation with the inflammatory mediator CX3CL1, typically expressed on neurons [135]; it is also produced by astrocytes and microglia in response to LPS, TNF, and IL-6 [136]. This emphasizes how HDPs are able to support communication between CNS cells and demonstrates the relationship between inflammation and iron metabolism in the CNS.

Finally, hepcidin release from astrocytes has been shown to induce neuronal apoptosis. Astrocyte-specific hepcidin knockdown mice had decreased levels of cleaved caspase 3 in neurons (a marker of increased apoptosis). The authors showed that the lack of hepcidin production by astrocytes protects neurons from inflammation-stimulated apoptosis by reducing neuronal iron concentration [137]. It is possible that hepcidin production in the CNS can induce apoptosis of other cells such as resident or infiltrating immune cells, which acts as an important immunomodulatory function.

### Neurodegeneration

It is well known that iron accumulation is a hallmark of neurodegenerative disease [138–140] and the implications of iron pathophysiology in neurological diseases have been reviewed extensively elsewhere [141–143]. Abnormal iron levels in the brain are involved in the formation of free radicals, which have been associated with oxidative damage and neuronal death [141]. Hepcidin regulates iron accumulation in microglia and astrocytes in diseases including AD, sporadic amyotrophic lateral sclerosis, Parkinson’s disease, and Sanfilippo syndrome [144–147]. Hepcidin levels increase with age in the rat in the cortex, striatum, hippocampus, and substantia nigra [148] and this was associated with increased pathological hallmarks of AD. However, a study showed that hepcidin was significantly reduced in post-mortem hippocampal lysates from patients with AD compared to healthy controls [149]. It is currently unclear why hepcidin expression is decreased in patients with AD [141, 149]. A reduction in hepcidin would lead to increased brain iron content which could partially explain the increased iron expression in the brains of patients with AD [141]. A recent study showed that overexpression of hepcidin in astrocytes of APP/PS1 mice significantly improved cognitive decline and partially reduced Aβ plaque formation in the cortex. They showed that overexpression of hepcidin reduced iron content in neurons which reduced iron accumulation-induced oxidative stress and cortex neuronal death [150]. Therefore, perhaps a similar mechanism is occurring in the human AD brain. Moreover, expression of the *HAMP* gene is upregulated in the spinal cord during the mouse model of multiple sclerosis, experimental autoimmune encephalomyelitis (EAE) [151], and in the cortex during the cuprizone model of demyelination [152]. Therefore, it is likely that dysregulated hepcidin leads to aberrant iron accumulation in multiple neurological diseases.

## A note on Amyloid-β

Interestingly, a review by Gosztyla and colleagues collated evidence indicating a relationship between Aβ peptides in AD and their proposed function as HDP [153]. Aβ peptides are considered the driving force leading to the development of AD; however, drugs targeting Aβ have not been successful in terms of reducing cognitive decline [154]. Moreover, one of the common adverse reactions of anti-Aβ treatment is increased incidence of infections, suggesting a link between Aβ and the fight against infection [153, 155, 156]. Specifically, Aβ has been suggested to function as an antimicrobial HDP. This was first proposed by Robinson and Bishop in the bioflocculant hypothesis, which stated that Aβ deposited by glial cells forms a web that surrounds neurons and protects them from pathogens [157]. They reference a study [158] showing intracerebroventricular injection of LPS promoted Aβ deposition in transgenic mice that overexpress mutated human APP, and suggest this provides evidence that Aβ can bind pathogens [157]. Since this was proposed, multiple studies have demonstrated evidence in support of the bioflocculant hypothesis. For example, Aβ can inhibit viral replication of influenza [159] and has shown to have antimicrobial activity against many microorganisms [153, 160] such as *Candida albicans, Streptococcus pneumoniae,* and *Pseudomonas aeruginosa*, and Aβ exhibited higher potency than cathelicidin for some pathogens [161]. Aβ oligomers bind AD-associated herpes simplex virus [162], and prevent the virus from entering cells [163] suggesting a protective role for Aβ in CNS innate immunity [164]. Intriguingly, amyloid fibrils are present on NETs [165] suggesting amyloid fibrils may be an important mediator of innate immunity. Finally, Aβ can be expressed by immortalized microglial cells following LPS exposure [166], demonstrating that it could be released from activated immune cells within the CNS in response to infection.

## A note on methodology

The central nervous system (CNS) was long considered an immune privileged site [167], separated from peripheral cells by the blood brain barrier. However, we now understand that immune cells do cross this barrier [168, 169] and patrol the brain and spinal cord during homeostasis, infection, and neurological disease. Indeed, granulocytes, including neutrophils, are present in the naïve mouse brain [170], comprising the third-largest tissue-resident leukocyte population in health [171]. In particular, neutrophils were noted in the dura mater, pia mater, and ependyma [171]. As neutrophils are one of the main cellular sources of HDP, storing cathelicidin and defensins in particular in abundance, neutrophil infiltration into the CNS is likely to contribute to HDP expression at this site.

HDP-secreting neutrophils also migrate into the CNS during neuroinflammation. Neutrophil infiltration into the spinal cord and brain tissue has been observed in mouse models of Alzheimer’s disease (AD) [59, 60] and multiple sclerosis (MS) [61, 62]. Importantly, cathelicidin-positive neutrophils are present in the spinal cord in EAE. Depletion of neutrophils or cathelicidin attenuates the development of EAE [43, 60, 62–64] and in AD models this can improve cognitive decline [65]. In humans, neutrophils are present in the CSF and in active lesions of neuromyelitis optica patients [66]. In other human neurological diseases, alterations to neutrophil populations have been noted.

This raises the possibility that if HDP are stored in infiltrating and/or resident neutrophils, or other granular cells, mRNA assays may not detect expression as the HDP is not actively being transcribed. Therefore, further studies are required which utilize a variety of complementary techniques to elucidate under what conditions HDP are expressed and by which cell types in the CNS. This is particularly true for understanding the role of neutrophil-derived and NET-coated HDP in the nervous system; we know neutrophils are present but unpicking their roles is technologically challenging.

In many of the papers described in this review, the cellular source of the HDP was not identified. In cells other than neutrophils, published single-cell RNA sequencing data from CNS studies can be exploited to pinpoint the expression of HDP. Likewise, as reagents improve, the cellular source of HDP in species other than humans and mice can be determined through co-localization with specific cell markers.

Finally, there are limitations in the availability of effective antibodies for detection the of HDP. For example, few reliable anti-HDP flow cytometry or immunofluorescence antibodies exist. Thus, the development of better reagents is essential to generate a complete map of HDP expression across the CNS across health, infection, and disease.

## Concluding remarks

Host defence peptides are not limited to mucosal sites or active infections; instead, we have shown that they are expressed in the central nervous system of a wide range of species. This broad expression—during infection and also sterile inflammation and neurodegeneration—suggests the peptides have multiple roles. We propose HDP have important functions not only as the first line of defence against pathogens but also as important immunomodulators. As the field develops and we understand the immunomodulatory roles of HDP in more detail, it is likely we will understand nervous system HDP to have roles we have not so far considered.
